# Mental health outcomes at intensive care unit discharge: prevalence, mediators and risk factors

**DOI:** 10.1186/s13613-025-01545-w

**Published:** 2025-09-25

**Authors:** Maryline Couette, Segolene Gendreau, Marie Charlotte Boishardy, Anne-Fleur Jean Baptiste, Paula Xavier, Keyvan Razazi, Romain Arrestier, Guillaume Carteaux, Nicolas De Prost, Stephane Mouchabac, Florian Ferreri, Armand Mekontso Dessap

**Affiliations:** 1 Service de Médecine Intensive Réanimation, AP-HP, Hôpitaux Universitaires Henri-Mondor, Créteil, F-94010 France; 2 Univ Paris Est Créteil, INSERM, IMRB, Créteil, F-94010 France; 3 Univ Paris Est Créteil, CARMAS, Créteil, F-94010 France; 4 Department of Psychiatry, Hôpital Saint-Antoine, AP-HP, Sorbonne Université, Paris, 75012 France; 5 iCRIN (Infrastructure for Clinical Research in Neurosciences), Brain Institute (ICM), Sorbonne Université, INSERM, CNRS, Paris, 75013 France

**Keywords:** Post intensive care syndrome, Psychological disorders, Acute stress disorder, Early psychological assessment, Trauma

## Abstract

**Background:**

Intensive Care Unit (ICU) patients often experience significant discomfort and distress due to both the medical environment and the nature of their stay. While long-term sequelae such as depression, anxiety, and post-traumatic stress are well-documented, few studies have examined psychological disorders present at the time of ICU discharge. Based on the model of Post-Intensive Care Syndrome, specifically the mental component (PICS-M), we defined DICS-M (Discharge Intensive Care Syndrome - Mental component). This study aimed to estimate the prevalence of psychological disorders at ICU discharge and to identify potential mediators and risk factors.

**Methods:**

We conducted a prospective observational study involving 243 patients admitted between January 2023 and April 2024.

**Results:**

The prevalence of DICS-M was 53% [95% CI: 46–59], with acute stress, anxiety, and depression observed in 37%, 36%, and 23% of patients, respectively. The analyses revealed an overlap among these psychological components. Peritraumatic distress acted as the main mediator of DICS-M. Univariate and multivariable analyses identified female gender and a history of psychiatric and cardiac conditions as risk factors of DICS-M.

**Conclusion:**

Psychological disorders are common at ICU discharge, mediated by peritraumatic distress, and associated with identifiable risk factors. These findings may help guide interventions to prevent long-term sequelae of ICU stays.

**Supplementary Information:**

The online version contains supplementary material available at 10.1186/s13613-025-01545-w.

## Introduction

Critically ill patients are exposed to a wide range of physical injuries, stressful events, and discomfort in the Intensive Care Unit (ICU) [1–4]. They are also at high risk of dying from vital organ failure. The ICU admission itself constitutes a major psychological stressor, often triggering fears of health deterioration and even imminent death. The combination of critical illness and the ICU environment aligns with the criteria for a traumatic event as defined by the DSM-5, which describes trauma as exposure to actual or threatened death, serious injury, or sexual violence [5].

A traumatic event can elicit two types of immediate or near-immediate reactions: (i) peritraumatic distress, assessed by the Peritraumatic Distress Inventory (PDI) [6], which refers to the “fear, helplessness, and horror” felt during or immediately after exposure to trauma, corresponding to criterion A2 of the DSM-5; (ii) peritraumatic dissociation, assessed using the Peritraumatic Dissociative Experiences Questionnaire (PDEQ) [7], which involves disturbances in time perception, spatial awareness, and self-identity. Both reactions are strong predictors of the long-term development of posttraumatic stress disorder (PTSD). PTSD, along with anxiety and depression, is one of the three disorders in the mental health component of post-intensive care syndrome (PICS-M) [8].

There is a relative paucity of studies examining psychological status on a shorter time scale at discharge from the ICU, particularly with regard to the gold standard scales for assessing trauma reactions: dissociation (PDEQ) and peritraumatic distress (PDI). The Haute Autorité de Santé in France advises clinicians to identify patients at risk of PICS-M at an early stage (Haute Autorité de Santé - Diagnostic et prise en charge des patients adultes avec un syndrome post-réanimation (PICS) et de leur entourage, s.d.). We hypothesized that a significant number of ICU survivors may already have psychological disorders at the time of ICU discharge, mediated by their reaction to perceived trauma during the ICU stay. We therefore defined DICS-M (Discharge Intensive Care Syndrome - Mental component) to describe psychological symptoms such as at least one or more mental health disorder at the time of ICU discharge including anxiety, depression and acute stress features identified at ICU discharge.

The primary objective of this observational study was to determine the prevalence of DICS-M at ICU discharge using psychotraumatic assessment tools. Additionally, we sought to identify potential mediators (notably, peritraumatic responses to perceived trauma) and risk factors associated with ICU stay and patient characteristics.

## Methods

### Patients and characteristics

Adult patients admitted to the medical ICU of Henri Mondor University Hospital between January 2023 and April 2024 were included in the study if they were hospitalized for at least 24 h and evaluated for mental health disorders at the time of ICU discharge. Patients were excluded if they did not understand written and/or spoken French, if they were deaf, demented, refused to participate, or had psychosis. In line with the literature on PICS [9–13], we chose to include patients with a history of psychiatric conditions, such as depression. However, we excluded patients with more severe psychiatric disorders or an active depressive state, defined as currently undergoing psychiatric treatment or when the psychiatric condition was the primary reason for ICU admission (e.g., suicidal behavior). The duties of the psychologist and a detailed description of the ICU unit at Henri Mondor can be found in the supplemental data.

Since 2023, a trained psychologist has aimed to routinely assess mental health disorders in the ICU and at discharge using four self-report questionnaires: the Peritraumatic Distress Inventory (PDI) and Peritraumatic Dissociative Experiences Questionnaire (PDEQ) for traumatic reactions, the Impact of Event Scale-Revised (IES-R) for acute stress, and the Hospital Anxiety and Depression Scale (HADS) for anxiety and depression. If psychological issues are identified at discharge, the psychologist provides brief psychoeducation on ICU-specific experiences, such as pain, loss of control, possible hallucinations, nightmares, and delusional memories. In-hospital follow-up is managed by the ward psychologist, with the option for the ICU psychologist to conduct a follow-up consultation after discharge if necessary.

### Outcomes and definitions

The primary outcome was the incidence of DICS-M, defined as the presence of at least one of the following at the time of ICU discharge: anxiety, depression, and acute stress. Anxiety and depression were assessed using the Hospital Anxiety and Depression Scale (HADS), which consists of a fourteen-item checklist divided into two subscales: anxiety (odd items) and depression (even items). Each item is scored on a 4-point frequency scale ranging from 0 to 3 [14]. The total score for each subscale ranges from 0 to 21, and a score of 8 or higher indicates suspicion of depression or anxiety.

Acute stress is characterized by intense stress that occurs at least 3 days but no more than 1 month after the traumatic event [15, 16].

The Impact of Event Scale-Revised (IES-R) is a brief self-report questionnaire developed by Weiss et al. [17]. It includes three symptom clusters: hyperarousal, intrusion, and avoidance, and is specifically designed to assess stress reactions following trauma. The IES-R is scored on a 5-point Likert-type scale ranging from 0 (not at all) to 4 (extremely), resulting in a total score ranging from 0 to 88. A score of 22 or higher indicates the presence of acute stress [17, 18].

The secondary objectives were to identify mediators and risk factors for DICS-M. For mediation, we tested two peritraumatic reactions (dissociation and distress, assessed by the Peritraumatic Dissociative Experiences Questionnaire [PDEQ] and the Peritraumatic Distress Inventory [PDI], respectively) as mediators of DICS-M following perceived trauma. Perceived trauma was defined according to the DSM-5 definition of trauma and, in the context of intensive care, refers to exposure to a threat of death or serious injury, inducing intense fear of dying and feelings of horror and helplessness [5].

The PDEQ is a 10-item self-report questionnaire designed to assess the degree of dissociation experienced during or immediately after trauma, including confusion, depersonalization, disruption of reality perception, impairment of temporal perception, and out-of-body experiences. It is scored on a 5-point Likert-type scale from 1 (not at all) to 5 (extremely true), resulting in a total score range from 10 to 50. A cut-off score of 15 or above indicates a dissociative response.

The PDI is a 13-item self-report questionnaire designed to assess emotional distress during and immediately after trauma. It is scored on a 5-point Likert-type scale from 0 (not at all true) to 4 (extremely true), resulting in a total score range from 0 to 52. A cut-off score of 15 or above indicates peritraumatic distress [6, 7].

The scales used are available in the supplemental material. To identify potential risk factors for DICS-M, we assessed the following patient and ICU characteristics: medical history, ICU admission severity score using the Simplified Acute Physiology Score (SAPS) 2 [19], organ function and support (hemodynamic, respiratory, and neurologic) at admission, and complications during the ICU stay.

### Statistical tests

Continuous data were expressed as median [interquartile range] and compared using the Mann-Whitney U test. Categorical variables, expressed as counts and percentages, were compared using the chi-squared test or Fisher’s exact test.

To examine the process linking perceived trauma to DICS-M, we evaluated dissociation and emotional distress as mediators of acute stress, anxiety, and depression. Mediation analysis involved performing successive regression models to assess the impact of each mediator on the relationship between perceived trauma (as the independent variable) and acute stress, anxiety, and depression, respectively [20].

To identify independent factors associated with the presence of DICS-M, significant or marginally significant bivariate risk factors (*p* < 0.25 using the above tests) were examined using univariate and multivariable backward stepwise logistic regression analysis. Coefficients were estimated using maximum likelihood. Model calibration was assessed using the Hosmer-Lemeshow goodness-of-fit statistic (a good fit was defined as a *p*-value > 0.05), and discrimination was evaluated by the area under the receiver operating characteristic curve (ROC-AUC), where a value of 1 indicates perfect discrimination and a value of 0.5 suggests chance alone.

Data were analyzed using Jamovi version 2.6 [21].

### Sample size justification

In accordance with the findings of the study conducted by Hatch et al. [22], which documented a 55% prevalence of the mental component of PICS at three months following ICU discharge, a sample size of 196 patients was deemed sufficient to estimate a prevalence of 50% of DICS-M with a confidence level of 95% and a marginal error of 7%. However, in order to ensure the robustness of our findings, we have decided to include a total of at least 200 patients in our study.

### Ethics

This study was reviewed and approved by the ethics committee of the SRLF-Société de Réanimation de Langue Française (CE-SRLF 25012). Patients and their relatives received an information letter given the observational nature of the study, as per French law.

## Results

### Patients characteristics

**Fig. 1:**
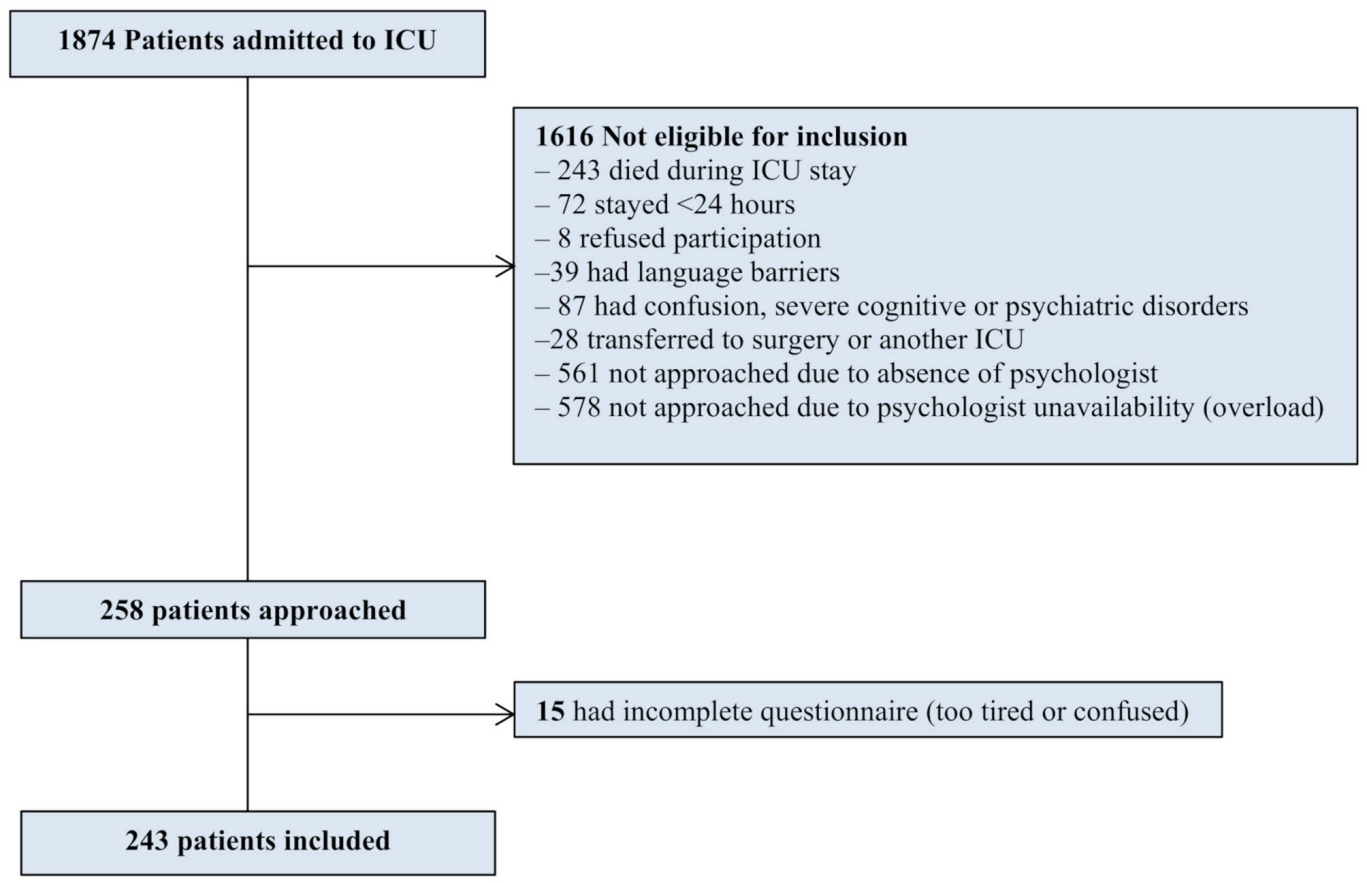
Flow chart *Categories are not mutually exclusive; a single patient may appear in more than one group

The selection process for the study is illustrated in the flowchart (Fig. 1.). A total of 1,874 patients were admitted to the ICU during the study period. Of these, 1,616 were deemed not eligible for inclusion based on predefined criteria: 243 patients died during their ICU stay, 72 stayed for less than 24 h, 8 refused to participate, 39 faced language barriers, and 87 presented with confusion or had severe cognitive or psychiatric disorders. It should be noted that these categories are not mutually exclusive, and a single patient may have met multiple exclusion criteria. Among the remaining patients, 258 were approached for participation, and ultimately, we enrolled 243 patients who responded to the questionnaires at ICU discharge. The median age was 50 [15–91] years and 132 (54%) patients were female. The median SAPS 2 score was 31 [3–98] and 88 (36%) of the patients had orotracheal intubation during ICU stay. Characteristics of the population, prevalence of perceived trauma, traumatic reactions and psychological disorders are reported in Tables 1 and 2.

**Table 1:** Prevalence of perceived trauma, traumatic reactions and psychological disorders

	*N*	Prevalence (*n*) [95%CI]
Trauma Event	243	61% (149) [55–67]
Dissociation	243	73% (177) [67–78]
Peritraumatic Distress	242	44% (106) [37–50]
Acute Stress	239	37% (88) [31–43]
Anxiety	242	36% (88) [30–43]
Depression	242	23% (56) [18–29]
DICS-M	243	53% (128) [46–59]

DICS-M, Discharge Intensive Care Syndrome – Mental component

**Table 2:** Characteristic of critically-ill patients, overall and depending on the presence of a DICS-M

Parameter	Available data	All patients (*n* = 243)	DICS-M (*n* = 128)	No DICS-M (*n* = 115)	*P* value
**PATIENT CHARACTERISTICS**					
Female gender	243	132 (54%)	80 (33%)	52 (21%)	0.007
Age (years)	243	50 [15–91]	52 [32–67]	51 [35–66]	0.888
Illness Severity (SAPS II)	238	31 [3–98]	30 [19–45]	31 [19–50]	0.700
**PAST MEDICAL HISTORY**					
Respiratory	243	64 (26%)	34 (14%)	30 (12%)	0.933
Cardiac	243	65 (27%)	42 (17%)	23 (9%)	0.024
Neurological	243	45 (18%)	25 (10%)	20 (8%)	0.668
Cirrhosis	243	9 (4%)	4 (2%)	5 (2%)	0.614
Cancer	243	25 (10%)	15 (6%)	10 (4%)	0.439
Hemopathy	243	8 (4%)	4 (2%)	4 (2%)	0.878
Sickle Cell Disease	243	68 (28%)	35 (14%)	33 (14%)	0.815
Chronic kidney failure	243	23 (9%)	13 (5%)	10 (4%)	0.698
Dialysis	243	5 (2%)	3 (1%)	2 (1%)	0.740
Depression/Anxiety	243	35 (14%)	28 (11%)	7 (3%)	< 0.001
Alcohol	243	31 (13%)	14 (6%)	17 (7%)	0.370
Drug addiction	243	8 (3%)	5 (2%)	3 (1%)	0.571
**ICU CONDITIONS**					
Renal replacement therapy	243	26 (11%)	13 (5%)	13 (5%)	0.773
Shock	243	85 (35%)	43 (18%)	42 (17%)	0.633
Orotracheal intubation	243	88 (36%)	43 (18%)	45 (18%)	0.370
OTI duration in days, intubated patients	88	5 [1–89]	5 [2–13]	6 [3–13]	0.293
Tracheostomy	243	15 (6%)	5 (2%)	10 (4%)	0.121
ECMO	243	10 (4%)	3 (1%)	7 (3%)	0.142
ARDS	243	27 (11%)	11 (4%)	16 (7%)	0.188
Continuous sedation*	243	83 (34%)	40 (16%)	43 (18%)	0.314
Sedation duration in days	83	3 [1–55]	3 [2–7]	3 [2–5]	0.710
Sufentanyl use	243	84 (35%)	42 (17%)	42 (17%)	0.507
Sufentanyl duration in days, all patients	239	0 [0–55]	0 [0–2]	0 [0–3]	0.314
Sufentanyl duration in days, patients receiving the drug	83	3 [1–55]	2 [2–7]	3 [2–9]	0.240
Ketamine use	240	34 (14%)	19 (8%)	15 (6%)	0.708
Lenght of stay in days	238	6 [1-111]	6 [3–10]	6 [4–11]	0.532

SAPS, simplified acute physiologic score; ICU, intensive care unit; OTI, orotracheal intubation; ECMO, extracorporeal membrane oxygenation; ARDS, acute respiratory distress syndrome; *propofol and/or midazolam

### DICS-M and its components

**Fig. 2:**
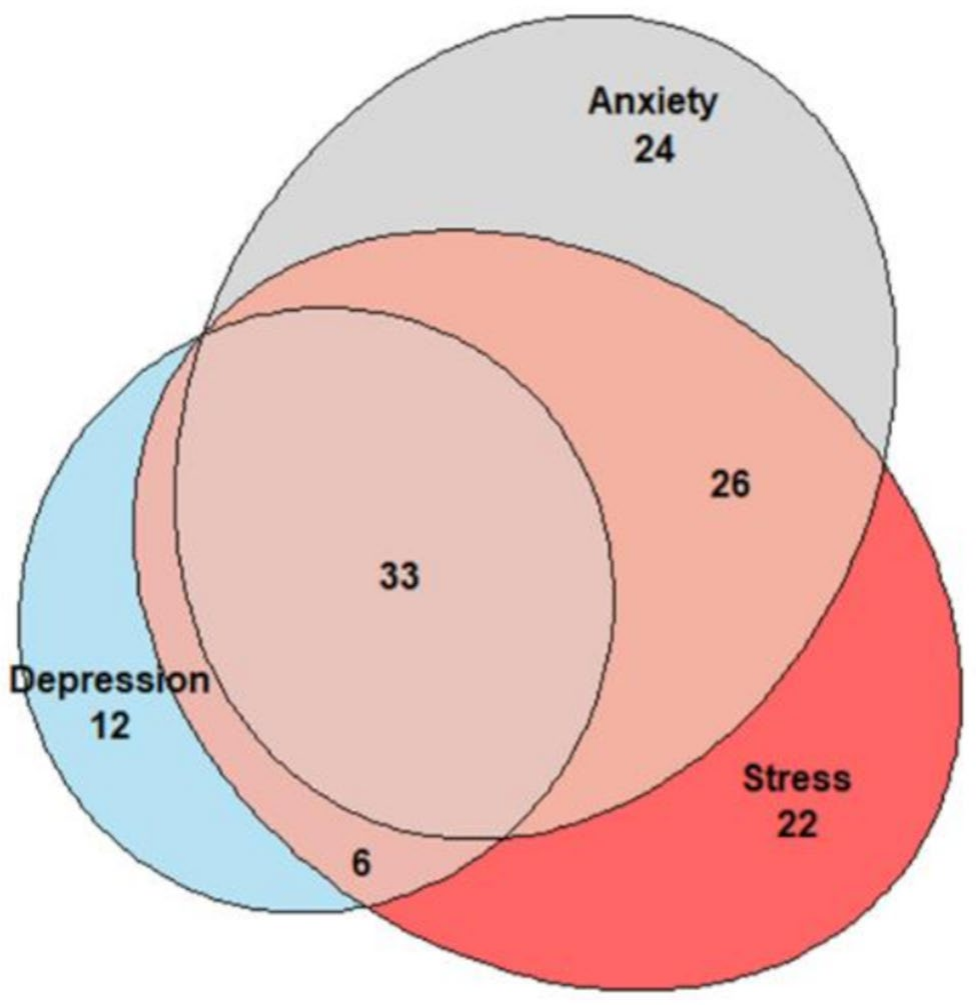
Euler diagram illustrating psychological outcomes at intensive care unit discharge among 128 patients diagnosed with DICS-M

At ICU discharge, the prevalence of DICS-M was 53% [95% CI: 46–59], while the prevalences of acute stress, anxiety and depression symptoms were 37% [95% CI: 31–43], 36% [95% CI: 30–43], and 23% [95% CI: 18–29], respectively. The Euler diagram showed a significant overlap between the three components of DICS-M (Fig. 2).

### Mediation of DICS-M

**Fig. 3:**
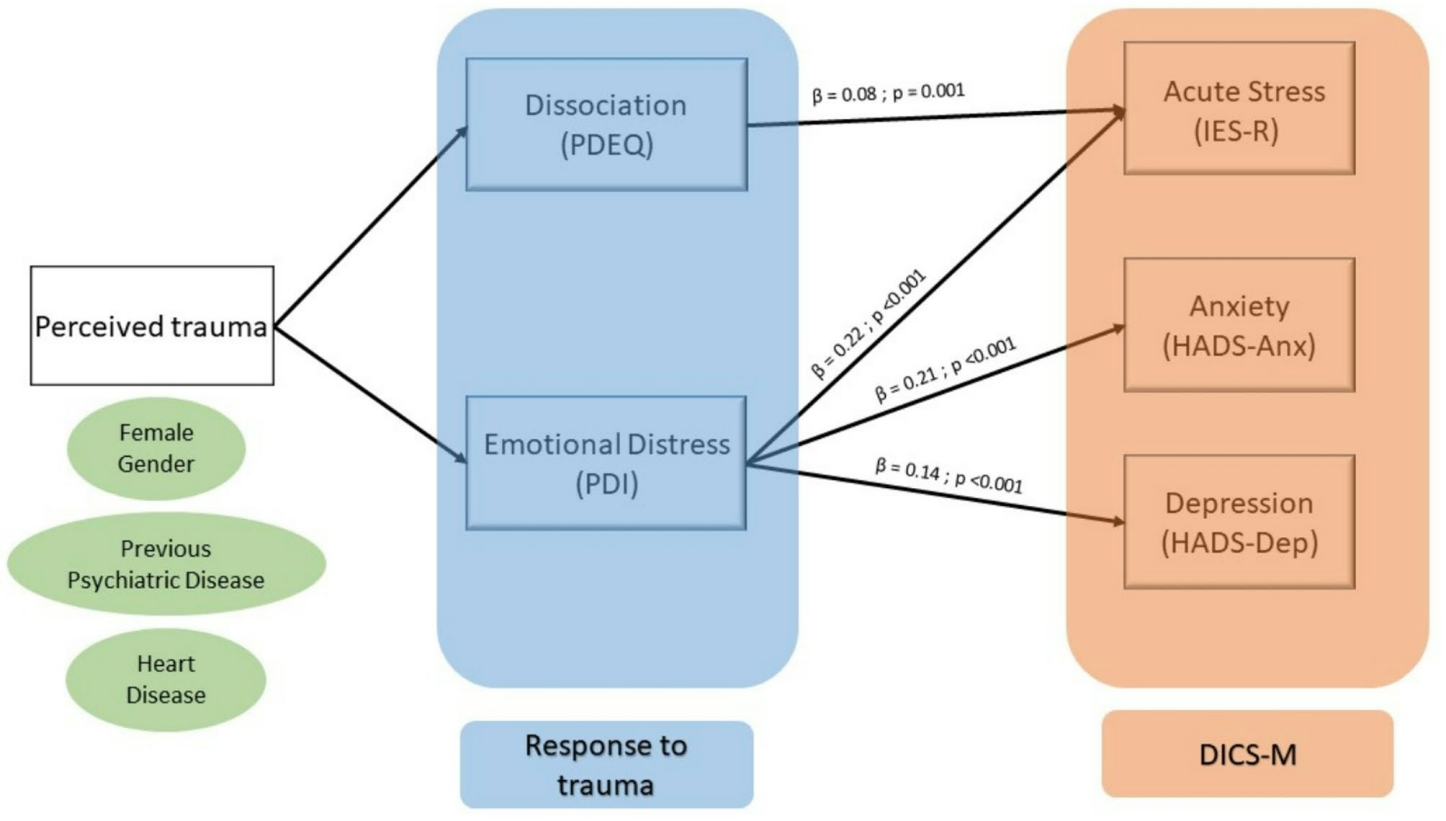
A general linear model was used to examine the relationship between perceived trauma and three psychological outcomes, mediated by two types of traumatic reactions: emotional peritraumatic distress and dissociation. The mediation model indicated that the indirect pathway from perceived trauma to each psychological outcome through emotional distress was statistically significant (*p* < 0.001). In this figure, are represented in blue and in green respectively the mediators and risk factors influencing the mental health component of discharge-intensive care syndrome

61% [95% CI: 55–67] of patients discharged from the ICU experienced a traumatic event. The prevalence of peritraumatic reactions was 73% [95% CI: 67–78] for dissociation and 44% [95% CI: 37–50] for emotional peritraumatic distress. The mediation model for DICS-M following perceived trauma yielded the following results: acute stress was significantly mediated by both emotional distress (β = 0.22, *p* < 0.001) and dissociation (β = 0.08, *p* = 0.001), whereas anxiety and depression were mediated only by emotional distress (β = 0.21, *p* < 0.001 and β = 0.14, *p* < 0.001, respectively) (Fig. 3, and Tables S1-S3 in the supplementary material).

### Risk factors for DICS-M

DICS-M was more frequent among female patients, those with a past medical history of cardiac disease, and those with a previous psychiatric disorder, as shown by both univariate and multivariable (Table 3) analyses.

**Table 3:** Factors associated with DICS-M by multivariable analysis

	Odds Ratio [95% Confidence Interval] by logistic regression	
Variable	Univariate	Multivariable
Demographics and past medical history		
Female gender	2.02 (1.21–3.37), *p* = 0.007	1.86 (1.07–3.24), *p* = 0.027
Cardiac disease	1.95 (1.09–3.51), *p* = 0.025	2.34 (1.25–4.40), *p* = 0.008
Previous psychiatric disease	4.32 (1.81–10.33), *p* = 0.001	4.33 (1.72–10.90), *p* = 0.002
ICU conditions		
Tracheostomy	0.43 (0.14–1.29), *p* = 0.131	0.25 (0.04–1.60), *p* = 0.144
ECMO	0.37 (0.09–1.47), *p* = 0.157	0.40 (0.08–1.93), *p* = 0.254
ARDS	0.58 (0.23–1.31), *p* = 0.192	0.77 (0.23–2.59), *p* = 0.678
Duration of invasive mechanical ventilation	1.01 (0.99–1.04), *p* = 0.308	0.99 (0.94–1.04), *p* = 0.678
Duration of sufentanyl infusion	1.01 (0.96–1.06), *p* = 0.663	0.97 (0.89–1.05), *p* = 0.431

The multivariable model did not show a good calibration as assessed by the Hosmer and Lemeshow goodness of fit test [χ^2^ (8 df) = 29.3, *p* < 0.001] but had a fair discrimination as assessed by the receiver operating characteristics curve [area under the curve of 0.70]

## Discussion

This is the first study to assess the mental health component of PICS early, at ICU discharge, with the following main findings: (i) half of ICU survivors leave the unit with DICS-M; (ii) the components of DICS-M (acute stress disorder, anxiety, and depression) significantly overlap and are primarily mediated by emotional peritraumatic distress, with a lesser contribution from dissociation; (iii) risk factors for DICS-M are largely related to patient characteristics, including female sex and a history of cardiac or psychiatric illness.

### Burden of DICS-M

Our study demonstrates a high prevalence of mental disorders at discharge, consistent with findings from most studies on the prevalence of PICS three months or more post-discharge. For instance, Hatch et al. reported a 55% prevalence of PICS-M at three months post-discharge, with 46% of patients experiencing anxiety, 40% depression, and 22% PTSD, assessed using the HADS scale and PCL-C [22]. Similarly, a recent synthesis of the literature highlights the significant burden of psychological sequelae following critical care discharge, with PICS-M prevalence rates ranging from 13 to 60% [23].

Anxiety disturbances, particularly acute stress and anxiety, exhibited the highest prevalence of psychological disorders in our study, affecting 37% and 36% of patients, respectively. These findings align with a recent study reporting a 43% prevalence of acute stress, assessed using the IES-R, within 1 to 8 days post-discharge [24].

Our study revealed a significant overlap among the three components of DICS-M, with anxiety disorders being the most prevalent. This overlap is consistent with findings in the PICS-M literature. Hatch et al. reported a similar overlap, with 18% of patients exhibiting depression, anxiety, and PTSD simultaneously, compared to 26% in our study at discharge [22]. Furthermore, a longitudinal study by Bienvenu et al. observed prolonged mental health disorder overlap, with 13% of patients experiencing all PICS-M components five years post-discharge from surgical and medical ICUs, assessed using the HADS and IES-R scales [25]. This overlap is unsurprising, as anxiety and depression frequently co-occur with PTSD [26].

### Mediators of DICS-M

Our mediation analysis clearly shows that peritraumatic distress, as measured by the PDI scale, is the only factor that simultaneously impacts acute stress, depression, and anxiety, whereas dissociation, as measured by the PDEQ, affects only acute stress. These findings suggest that peritraumatic distress represents the primary trauma response most likely to predict psychological disturbances at ICU discharge. To our knowledge, this is the first study to establish the role of trauma response assessment tools in identifying psychological issues at the time of ICU discharge. However, previous research has already emphasized the predictive value of the PDI scale for the later development of PTSD and other psychological consequences in patient populations at risk of ICU admission, such as those with acute coronary syndrome or injuries from motor vehicle accidents [27–29]. The PDI scale may more accurately capture the patient’s subjective experience and distress compared to the PDEQ scale, which might be influenced by the effects of ICU-related interventions, potentially inducing phenomena such as derealization, dissociation from the body, and, in some cases, “out-of-body” experiences.

### Risk factors for DICS-M

We identified a history of psychiatric illness as a significant risk factor for DICS-M, consistent with existing literature on PICS-M and its components [9–13]. Additionally, our study suggested that being female may be a vulnerability factor associated with psychological issues at ICU discharge. A recent WHO study surveying 68,894 individuals across 24 countries found that women were more likely than men to develop PTSD following similar traumatic events [30]. This study also highlighted that prior exposure to traumatic events or psychological vulnerability could contribute to the development of PTSD. It is therefore unsurprising that these factors are associated with psychological disorders. Although the survey did not specifically address trauma related to ICU experiences, it emphasized that events such as rape and sexual assault are the most significant contributors to PTSD. In the ICU setting, the integrity of the body is compromised by the need for invasive care and organ support machines. Additionally, the lack of privacy and autonomy may exacerbate psychological distress.

Our study also found that a history of cardiac disease was associated with DICS-M, particularly acute stress symptoms. While this association is noteworthy, its underlying mechanisms remain speculative. Some studies have suggested that exposure to adversity in childhood or adulthood may influence the development of both psychiatric and cardiovascular conditions [31, 32]. In such contexts, intense and chronic stress may lead to prolonged activation of stress-response systems, including sustained secretion of stress hormones and systemic inflammation, both of which are implicated in increased vulnerability to cardiovascular and psychological disorders [33]. This potential link should be considered exploratory and interpreted with caution.

### Clinical implications

The high prevalence of DICS-M and its components, affecting most ICU survivors at discharge, suggests that it should be considered a major public health priority. Identifying and managing patients with DICS-M could help reduce the overall sequelae related to ICU stays. Further research is needed to clarify the evolution of DICS-M and its relationship with PICS-M. Establishing such a link would emphasize the necessity for systematic early detection and intervention. If such a relationship exists, early identification of patients with DICS-M could be a critical step in the prevention of PICS-M. In line with health authority recommendations for early screening and the use of appropriate assessment tools, our study demonstrates that it is indeed feasible to quickly identify patients at risk of DICS-M by considering demographics, medical history, and prior psychiatric or cardiac conditions. Additionally, both psychotraumatic response assessment scales appear valuable in predicting acute stress and anxiety-depressive disorders. However, time constraints associated with conducting such screening in the ICU setting should be acknowledged.

Studies have shown that early interventions can enhance patient well-being and alleviate discomfort and stress in the ICU. Examples include physical rehabilitation care [34] and other relaxation techniques such as hypnosis [35, 36], music therapy [37], and meditative virtual reality [38]. Recognizing risk factors for DICS-M could help identify patients who may benefit from targeted stress-reduction care during their stay, allowing for personalized interventions tailored to individual medical backgrounds and demographic factors.

Given that acute stress disorder is often a natural reaction that can subside on its own, studies assessing the protective role of resilience could be valuable for early detection and for identifying coping mechanisms. Existing research highlights resilience as a crucial factor in psychological recovery from ICU-related trauma [39].

### Strengths and limitations

Strengths of our study include its prospective design, as well as the detailed psychological assessment conducted at ICU discharge for evaluating outcomes and potential mediators. However, our study also has several limitations. An important limitation is that, the effects of ICU-specific factors were not thoroughly collected in this study. Consequently, an association between these factors and the variables of interest cannot be ruled out, particularly since previous research has highlighted the impact of sedation on delirium [12, 40, 41]. It should be noted that the CAM-ICU was not systematically used for delirium assessment in this study, which represents a limitation. This state of confusion is exacerbated by the use of physical restraints [42], which were not accounted for in our dataset. Both confusion and physical restraints are known to induce significant psychological distress and are predictive of future post-traumatic stress [42]. Despite the lack of significant associations between ICU-related factors and DICS-M in our study, further research with more detailed phenotyping is warranted. Future studies should aim to incorporate granular data on sedation and analgesic dosages, the burden of organ support, use of physical restraints, and incidence of delirium to refine our understanding of these associations.

Another challenge was the impossibility to systematically assess all patients at discharge due to unplanned discharges or discharges occurring when the psychologist was unavailable. This limitation may introduce selection bias, impacting the representativeness of our sample and, consequently, the external validity of our study. ICU-associated psychological disorders are multifactorial, resulting from a combination of pre-existing vulnerabilities and the extreme stress of critical illness. Unlike some previous studies, we did not observe a significant association between ICU-related conditions, such as acute respiratory distress syndrome (ARDS) or septic shock, and the subsequent development of DICS-M. However, it is possible that patients with the most severe conditions were underrepresented in our sample due to selection bias, which may have influenced these results.

Another limitation of our study is that screening tools cannot be used to establish a clinical diagnosis of depression, since this requires a depressive state that has been present for at least 15 days. The HADS scale is designed to detect symptoms of depression and anxiety over the last seven days, so it does not allow us to determine whether the patient is experiencing a new anxiety-depression syndrome or an exacerbation of a pre-existing disorder. Nevertheless, recent studies have shown that the HADS is a robust clinical tool for physicians practicing in non-psychiatric departments to use for detecting depression. It is not always possible to conduct a rigorous clinical interview with a psychiatrist in an intensive care unit, and it is obvious that a screening tool cannot replace this [43, 44].

A further concern is that, the history of psychiatric disease may be underreported if not documented in medical records or disclosed by the patient. Information regarding past trauma or a history of psychiatric illness may be incomplete, as a comprehensive assessment was not always feasible in the context of critical care.

Future studies should consider systematic psychiatric screening methods to enhance the reliability of patient history data. The absence of post-discharge follow-up prevents us from assessing the persistence, resolution, or progression of psychological symptoms over time. As a result, we cannot determine whether these ICU-associated mental health disorders evolve into a chronic condition consistent with PICS-M.”

## Conclusion

In conclusion, this study highlights the high prevalence of psychological issues in ICU survivors at discharge. We identified peritraumatic distress, and to a lesser extent dissociation, as mediators, along with patient demographics (female sex) and past medical history (cardiac or psychiatric disease) as risk factors. These findings may facilitate the early identification and prevention of psychological sequelae in ICU survivors.

## Supplementary Information

Below is the link to the electronic supplementary material.

Supplementary Material 1

## Data Availability

The datasets used and/or analysed during the current study are available from the corresponding author on reasonable request.
