# Supplementary material for: Mental health outcomes at intensive care unit discharge: prevalence, mediators and risk factors
Source: Ann Intensive Care. 2025 Sep 25;15:138. doi: 10.1186/s13613-025-01545-w (PMC12463781; doi:10.1186/s13613-025-01545-w)
Supplement: Supplementary file 1 — Supplementary Material 1 [file 13613_2025_1545_MOESM1_ESM.docx]

**Mental health outcomes at intensive care unit discharge: prevalence, mediators and risk factors**

Maryline Couette^*1,2,^ Segolene Gendreau^1,2,3^, Marie Charlotte Boishardy^1^, Anne-Fleur Jean Baptiste^1^, Paula Xavier^1^, Keyvan Razazi^1,2,3^, Romain Arrestier^1,2,3^, Guillaume Carteaux^1,2,3^, Nicolas De Prost^1,2,3^, Stephane Mouchabac^4,5^, Florian Ferreri^4,5^, Armand Mekontso Dessap^1,2^.

Supplemental data

# Table S1. Mediation analysis table for indirect and total effects on acute stress disorder.

| Indirect and Total Effects | | | | | | | | |
| --- | --- | --- | --- | --- | --- | --- | --- | --- |
|  | | | | **95% C.I. (a)** | |  | | |
| **Type** | **Effect** | **Estimate** | **SE** | **Lower** | **Upper** | **β** | **z** | **p** |
| **Indirect** | **Trauma Event1 ⇒ PDEQ ⇒ IES-R** | 3.111 | 0.969 | 1.211 | 5.010 | 0.0862 | 3.21 | 0.001 |
|  | **Trauma Event1 ⇒ PDI ⇒ IES-R** | 7.999 | 1.424 | 5.208 | 10.790 | 0.2215 | 5.62 | <.001 |
| **Component** | **Trauma Event1 ⇒ PDEQ** | 7.768 | 1.146 | 5.522 | 10.015 | 0.4022 | 6.78 | <.001 |
|  | **PDEQ ⇒ IES-R** | 0.400 | 0.110 | 0.185 | 0.616 | 0.2142 | 3.64 | <.001 |
|  | **Trauma Event1 ⇒ PDI** | 8.787 | 1.165 | 6.504 | 11.070 | 0.4393 | 7.54 | <.001 |
|  | **PDI ⇒ IES-R** | 0.910 | 0.108 | 0.698 | 1.122 | 0.5043 | 8.42 | <.001 |
| **Direct** | **Trauma Event1 ⇒ IES-R** | 3.033 | 1.903 | -0.697 | 6.762 | 0.0840 | 1.59 | 0.111 |
| **Total** | **Trauma Event1 ⇒ IES-R** | 13.975 | 2.150 | 9.760 | 18.190 | 0.3882 | 6.50 | <.001 |
| *Note.* Confidence intervals computed with method: Standard (Delta method) | | | | | | | | |
| *Note.* Betas are completely standardized effect sizes | | | | | | | | |

# Table S2. Mediation analysis table for indirect and total effects on anxiety

| Indirect and Total Effects | | | | | | | | |  |  |
| --- | --- | --- | --- | --- | --- | --- | --- | --- | --- | --- |
|  | | | | **95% C.I. (a)** | |  | | |  |  |
| **Type** | **Effect** | **Estimate** | **SE** | **Lower** | **Upper** | **β** | **z** | **p** | | |
| **Indirect** | **Trauma Event1 ⇒ PDEQ ⇒ HADS-Anx** | 0.3066 | 0.2801 | -0.2423 | 0.856 | 0.03104 | 1.0947 | 0.274 | |  |
|  | **Trauma Event1 ⇒ PDI ⇒ HADS-Anx** | 2.0643 | 0.4087 | 1.2633 | 2.865 | 0.20896 | 5.0509 | <.001 | |  |
| **Component** | **Trauma Event1 ⇒ PDEQ** | 7.8439 | 1.1436 | 5.6025 | 10.085 | 0.40414 | 6.8591 | <.001 | |  |
|  | **PDEQ ⇒ HADS-Anx** | 0.0391 | 0.0352 | -0.0300 | 0.108 | 0.07680 | 1.1090 | 0.267 | |  |
|  | **Trauma Event1 ⇒ PDI** | 8.7355 | 1.1694 | 6.4435 | 11.027 | 0.43360 | 7.4700 | <.001 | |  |
|  | **PDI ⇒ HADS-Anx** | 0.2363 | 0.0345 | 0.1688 | 0.304 | 0.48192 | 6.8556 | <.001 | |  |
| **Direct** | **Trauma Event1 ⇒ HADS-Anx** | 0.0511 | 0.6098 | -1.1440 | 1.246 | 0.00517 | 0.0838 | 0.933 | |  |
| **Total** | **Trauma Event1 ⇒ HADS-Anx** | 2.3351 | 0.6188 | 1.1223 | 3.548 | 0.23620 | 3.7736 | <.001 | |  |
| *Note.* Confidence intervals computed with method: Standard (Delta method) | | | | | | | | |  |  |
| *Note.* Betas are completely standardized effect sizes | | | | | | | | |  |  |

# Table S3. Mediation analysis table for indirect and total effects on depression.

| Indirect and Total Effects | | | | | | | | | | | |
| --- | --- | --- | --- | --- | --- | --- | --- | --- | --- | --- | --- |
|  | | | | | **95% C.I. (a)** | | |  | | | |
| **Type** | **Effect** | **Estimate** | **SE** | **Lower** | | **Upper** | **β** | | **z** | **p** | |
| **Indirect** | **Trauma Event1 ⇒ PDEQ ⇒ HADS-Dep** | 0.2973 | 0.2760 | -0.2436 | | 0.838 | 0.0333 | | 1.077 | 0.281 |  |
|  | **Trauma Event1 ⇒ PDI ⇒ HADS-Dep** | 1.2671 | 0.3419 | 0.5970 | | 1.937 | 0.1417 | | 3.706 | <.001 |  |
| **Component** | **Trauma Event1 ⇒ PDEQ** | 7.8439 | 1.1436 | 5.6025 | | 10.085 | 0.4041 | | 6.859 | <.001 |  |
|  | **PDEQ ⇒ HADS-Dep** | 0.0379 | 0.0347 | -0.0302 | | 0.106 | 0.0823 | | 1.091 | 0.275 |  |
|  | **Trauma Event1 ⇒ PDI** | 8.7355 | 1.1694 | 6.4435 | | 11.027 | 0.4336 | | 7.470 | <.001 |  |
|  | **PDI ⇒ HADS-Dep** | 0.1451 | 0.0340 | 0.0785 | | 0.212 | 0.3268 | | 4.269 | <.001 |  |
| **Direct** | **Trauma Event1 ⇒ HADS-Dep** | 0.1438 | 0.6011 | -1.0343 | | 1.322 | 0.0161 | | 0.239 | 0.811 |  |
| **Total** | **Trauma Event1 ⇒ HADS-Dep** | 1.7191 | 0.5636 | 0.6145 | | 2.824 | 0.1928 | | 3.050 | 0.002 |  |
| *Note.* Confidence intervals computed with method: Standard (Delta method) | | | | | | | | | | | |
| *Note.* Betas are completely standardized effect sizes | | | | | | | | | | | |

# Supplemental methods: Detailed description of Henri Mondor medical ICU unit and duties of the psychologist

The unit comprises 43 beds: 25 Level 3 beds (Full ICU / Critical Care), 12 Level 2 beds (High Dependency Unit, HDU, or Step-Down ICU), and 6 beds dedicated to mechanical ventilation weaning. The medical and paramedical staff includes 25 physicians, 79 nurses, 53 nursing assistants, 4 physiotherapists, 4 secretaries, 1 social worker, 1 psychologist, and 1 receptionist. The paramedical team works in 12-hour shifts to ensure continuous, round-the-clock care.

The ICU is open to relatives 24 hours a day, although they are encouraged to respect the patient’s need for rest, particularly during nighttime hours. In exceptional circumstances, nighttime visits are permitted. To preserve a calm environment, visits are limited to three people at a time. Children under the age of 15 may visit after an assessment and discussion with the psychologist and the care team.

Families of intubated and mechanically ventilated patients are offered a diary, although this practice is not systematic. A welcome booklet is also provided to explain the functioning of the unit, present the team, and inform families about the available support. This booklet includes a section on post-ICU care, with information for patients and relatives on how to remain in contact after discharge.

The ICU has one full-time psychologist. Their responsibilities include patient support, family support, staff debriefing following critical incidents, follow-up consultations at three months post-ICU, psychological follow-up for selected patients, bereavement support, and participation in clinical research.

# Questionnaires

## PDEQ

|  | Pas du tout vrai | Un peu vrai | Plutôt vrai | Très vrai | Extrê meme  nt vrai |
| --- | --- | --- | --- | --- | --- |
| 1. Il y a eu des moments où j’ai perdu le fil de ce qui se passait – j’étais complètement déconnecté(e) ou, d’une certaine façon, j’ai senti que je ne faisais pas partie de ce qui se passait |  |  |  |  |  |
| 2. Je me suis retrouvé(e) sur le "pilote automatique", je me suis mis(e) à faire des choses que, je l’ai réalisé plus tard, je n'avais pas activement décidé de faire |  |  |  |  |  |
| 3. Ma perception du temps a changé; les choses avaient l’air de se dérouler au ralenti |  |  |  |  |  |
| 4. Ce qui se passait me semblait irréel, comme si j’étais dans un rêve, ou au cinéma, ou en train de jouer un rôle |  |  |  |  |  |
| 5. C'est comme si j'étais le ou la spectateur(trice) de ce qui m'arrivait, comme si je flottais au-dessus de la scène et l'observais de l'extérieur |  |  |  |  |  |
| 6. Il y a eu des moments où la perception que j'avais de mon corps était distordue ou changée. Je me sentais déconnecté(e) de mon propre corps, ou bien il me semblait plus grand ou plus petit que d'habitude |  |  |  |  |  |
| 7. J'avais l'impression que les choses qui arrivaient aux autres m'arrivaient à moi aussi, comme par exemple être en danger alors que je ne l'étais pas |  |  |  |  |  |
|  |  |  |  |  |  |

|  | Pas du tout vrai | Un peu vrai | Plutôt vrai | Très vrai | Extrê meme  nt vrai |
| --- | --- | --- | --- | --- | --- |
| 8. J'ai été surpris(e) de constater après coup que plusieurs choses s'étaient produites sans que je m'en rende compte, des choses que j'aurais habituellement remarquées |  |  |  |  |  |
| 9. J’étais confus(e) :c’est-à-dire que par moment j’avais de la difficulté à  comprendre ce qui se passait vraiment |  |  |  |  |  |
| 10. J’étais désorienté(e) : c’est-à-dire que par moment j’étais incertain(e) de l'endroit où je me trouvais, ou de l’heure qu’il était |  |  |  |  |  |

## PDI

|  | Pas du tout vrai | Un peu vrai | Plutôt vrai | Très vrai | Extrêmemen  vrai |
| --- | --- | --- | --- | --- | --- |
| 1. Je ressentais de l’impuissance |  |  |  |  |  |
| 2. Je ressentais de la tristesse et du chagrin |  |  |  |  |  |
| 3. Je me sentais frustré(e) et en colère |  |  |  |  |  |
| 4. J’avais peur pour ma propre sécurité |  |  |  |  |  |
| 5. Je me sentais coupable |  |  |  |  |  |
| 6. J’avais honte de mes réactions émotionnelles |  |  |  |  |  |
| 7. J’étais inquiet pour la sécurité des autres |  |  |  |  |  |
| 8. J’avais l’impression que j’allais perdre le contrôle de mes émotions |  |  |  |  |  |
| 9. J’avais envie d’uriner et d’aller à la selle |  |  |  |  |  |
| 10. J’étais horrifié(e) |  |  |  |  |  |
| 11. J’avais des réactions physiques comme des sueurs, des tremblements et des palpitations |  |  |  |  |  |
| 12. Je sentais que je pourrais m’évanouir |  |  |  |  |  |
| 13. Je pensais que je pourrais mourir |  |  |  |  |  |

## Impact of Event Scale-Revised

**INSTRUCTIONS : 1.** Veuillez lire chaque item et indiquer à quel point vous avez été bouleversé(e) par chacune de ses difficultés au cours des 7 derniers jours concernant votre événement.

Dans quelles mesure avez-vous été affecté(e) ou bouleversé(e) par ces difficultés?

|  | Pas du tout | Un peu | Moyen nement | Passab lement | Extrême -ment |
| --- | --- | --- | --- | --- | --- |
| 1. Tout rappel de l’événement ravivait mes sentiments en rapport avec celui-ci. | 1 | 2 | 3 | 4 | 5 |
| 2. Je me réveillais la nuit. | 1 | 2 | 3 | 4 | 5 |
| 3. Différentes choses me faisaient y penser. | 1 | 2 | 3 | 4 | 5 |
| 4. Je me sentais irritable et en colère. | 1 | 2 | 3 | 4 | 5 |
| 5. Quand j’y repensais ou qu’on me le rappelait, j’évitais de me laisser bouleverser. | 1 | 2 | 3 | 4 | 5 |
| 6. Sans le vouloir j’y repensais. | 1 | 2 | 3 | 4 | 5 |
| 7. J’avais l’impression que rien n’était vraiment arrivé ou que cela n’était pas réel. | 1 | 2 | 3 | 4 | 5 |
| 8. Je me suis tenu(e) loin de ce qui me faisait y penser. | 1 | 2 | 3 | 4 | 5 |
| 9. Des images de l’événement surgissaient dans ma tête. | 1 | 2 | 3 | 4 | 5 |
| 10. J’étais nerveux (se) et je sursautais facilement. | 1 | 2 | 3 | 4 | 5 |
| 11. J’essayais de ne pas y penser. | 1 | 2 | 3 | 4 | 5 |
| 12. J’étais conscient(e) d’avoir encore beaucoup d’émotions à propos de l’événement, mais je n’y ai pas fait face. | 1 | 2 | 3 | 4 | 5 |
|  |  |  |  |  |  |

|  | Pas du tout | Un peu | Moyenn ement | Passab lement | Extrême ment |
| --- | --- | --- | --- | --- | --- |
| 13. Mes sentiments à propos de l’événement étaient comme figés. | 1 | 2 | 3 | 4 | 5 |
| 14. Je me sentais et je réagissais comme si j’étais encore dans l’événement. | 1 | 2 | 3 | 4 | 5 |
| 15. J’avais du mal à m’endormir. | 1 | 2 | 3 | 4 | 5 |
| 16. J’ai ressenti des vagues de sentiments intenses à propos de l’événement. | 1 | 2 | 3 | 4 | 5 |
| 17. J’ai essayé de l’effacer de ma mémoire. | 1 | 2 | 3 | 4 | 5 |
| 18. J’avais du mal à me concentrer. | 1 | 2 | 3 | 4 | 5 |
| 19. Ce qui me rappelait l’événement me causait des réactions physiques telles que des sueurs, des difficultés à respirer, des nausées ou des palpitations. | 1 | 2 | 3 | 4 | 5 |
| 20. J’ai rêvé à l’événement. | 1 | 2 | 3 | 4 | 5 |
| 21. J’étais aux aguets et sur mes gardes. | 1 | 2 | 3 | 4 | 5 |
| 22. J’ai essayé de ne pas en parler. | 1 | 2 | 3 | 4 | 5 |

## *Hospital Anxiety and Depression Scale*

Dans la série de questions ci-dessous, cochez la réponse qui exprime le mieux ce que vous avez éprouvé au cours de la semaine qui vient de s’écouler. Ne vous attardez pas sur la réponse à faire : votre réaction immédiate à chaque question fournira probablement une meilleure indication de ce que vous éprouvez, qu’une réponse longuement méditée.

| **Score** | **Anxiété** | **Score** | **Dépression** |
| --- | --- | --- | --- |
| 3  2  1  0 | Je me sens tendu ou énervé :   - la plupart du temps - souvent - de temps en temps - jamais | 0  1  2  3 | Je prends plaisir aux mêmes choses qu’autrefois   - oui, tout autant - pas autant - un peu seulement - presque plus |
| 3  2  1  0 | J’ai une sensation de peur comme si quelque chose d’horrible allait m’arriver   - oui, très nettement - oui, mais ce n’est pas grave - un peu, mais cela ne m’inquiète pas - pas du tout | 0  1  2  3 | Je ris facilement et vois le bon côté des choses   - autant que par le passé - plus autant qu’avant - vraiment moins qu’avant - plus du tout |
| 3  2  1  0 | Je me fais du souci :  ❒très souvent  ❒assez souvent  ❒occasionnellement  ❒très occasionnellement | 3  2  1  0 | Je suis de bonne humeur :  ❒jamais  ❒rarement  ❒assez souvent  ❒la plupart du temps |
| 0  1  2  3 | Je peux rester tranquillement assis à ne rien faire et me sentir décontracté :  ❒oui, quoi qu’il arrive  ❒oui, en général  ❒rarement  ❒jamais | 3  2  1  0 | J’ai l’impression de fonctionner au ralenti :  ❒presque toujours  ❒très souvent  ❒parfois  ❒jamais |
| 0  1  2  3 | J’éprouve des sensations de peur et j’ai l’estomac noué :  ❒jamais  ❒parfois  ❒assez souvent  ❒très souvent | 3  2  1  0 | Je ne m’intéresse plus à mon apparence :   - plus du tout - je n’y accorde pas autant d’attention que je le devrais - il se peut que je n’y fasse plus autant attention - j’y prête autant d’attention que par le passé |
| 3  2  1  0 | J’ai la bougeotte et n’arrive pas à tenir en place :  ❒oui, c’est tout à fait le cas  ❒un peu  ❒pas tellement  ❒pas du tout | 0  1  2  3 | Je me réjouis d’avance à l’idée de faire certaines choses :  ❒autant qu’auparavant  ❒un peu moins qu’avant  ❒bien moins qu’avant  ❒presque jamais |
| 3  2  1  0 | J’éprouve des sensations soudaines de panique :  ❒vraiment très souvent  ❒assez souvent  ❒pas très souvent  ❒jamais | 0  1  2  3 | Je peux prendre plaisir à un bon livre ou à une bonne émission radio ou de télévision :  ❒souvent  ❒parfois  ❒rarement  ❒très rarement |
|  | ☜ **Total du score pour l’anxiété** |  | ☜ **Total du score pour la dépression** |

Chaque réponse correspond à un chiffre. En additionnant ces chiffres, on obtient un score total par colonne (anxiété et dépression). Si le score d’une colonne est supérieur ou égal à 11, cela signifie que vous souffrez d’anxiété ou de dépression (selon la colonne concernée).
